# ﻿A new species and new combination in basally flowering Vietnam Costaceae

**DOI:** 10.3897/phytokeys.190.76494

**Published:** 2022-02-25

**Authors:** Jana Leong-Škorničková, Alžběta Böhmová, Hữu Đăng Trần

**Affiliations:** 1 The Herbarium, Singapore Botanic Gardens, 1 Cluny Road, 259569, Singapore, Singapore The Herbarium, Singapore Botanic Gardens Singapore Singapore; 2 Department of Botany, Faculty of Science, Charles University, Benátská 2, CZ-128 01, Prague, Czech Republic Charles University Prague Czech Republic; 3 Department of Botany, National Museum in Prague, Cirkusová 1740, CZ-193 00, Prague, Czech Republic Department of Botany, National Museum in Prague Prague Czech Republic; 4 Becamex Institute of Research and Development, Becamex IDC Corp., 08, Hùng Vương St., Hòa Phú Ward, Thủ Dầu Một City, Bình Dương Province, Vietnam. Southern Institute of Ecology, Vietnam Academy of Science and Technology Ho Chi Minh City Vietnam; 5 Southern Institute of Ecology, Vietnam Academy of Science and Technology, Hồ Chí Minh City, Vietnam Becamex Institute of Research and Development, Becamex IDC Corp. Thủ Dầu Một City Vietnam

**Keywords:** Asia, *Cheilocostuscandidus*, *Cheilocostustonkinensis*, *Costus*, *Hellenia*, lectotype, spiral gingers

## Abstract

*Cheilocostuscandidus***sp. nov.** (Costaceae), a basally flowering spiral ginger with cream white flowers from southern Vietnam, is described and illustrated here. A new combination, *Cheilocostustonkinensis* (Gagnep.) Škorničk., is proposed here and the lectotype is designated. A key to *Cheilocostus* in Vietnam is included.

## ﻿Introduction

The family Costaceae, with well-developed and sometimes branched true stems and leaves spirally arranged in monophyllous phyllotaxy, is easy to recognise within the Zingiberales order (see, for example, Kirchoff and Rutishauser (1990)). Based on recent phylogenetic studies, seven genera amounting to approximately 140 species are recognised in Costaceae (Specht and Stevenson 2006). The majority of this diversity is confined to African and New World tropics (Specht and Stevenson 2006, Maas-van de Kamer et al. 2016), while only a handful of species are considered native to Asia.

**Figure 1.**
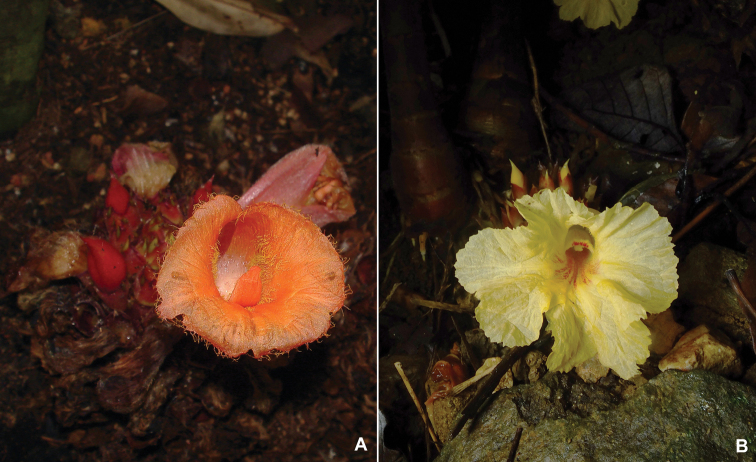
**A***Cheilocostusglobosus*, Singapore **B***Cheilocostustonkinensis* (Gagnep.) Škorničk., Vietnam, photographed at the type locality. Photo by Jana Leong-Škorničková.

During this recent generic re-assessment of Costaceae, the best known and pantropically widespread Asian member of the genus, *Costusspeciosus* J. König, as well as the other three Asian species (*C.lacerus* Gagnep., *C.globosus* Blume and *C.sopuensis* Maas & H. Maas), were transferred into the newly-established *Cheilocostus* (Specht and Stevenson 2006). The study, however, has not addressed the transfer of *Costustonkinensis* Gagnep., possibly due to Maas (1979) briefly expressing a concern that 11 species described from various parts of Asia might all be mere variants of *Cheilocostusglobosus* complex. While we fully agree that basally flowering Asian Costaceae are badly in need of a revision, our studies over the last decade on flowering material in SE Asia and Indochina, in particular, support the conclusion of Wu and Larsen (2000) that *Costustonkinensis*, described from N. Vietnam, certainly represent a distinct species from *Ch.globosus* (see Fig. 1).

Specht and Stevenson (2006) overlooked the availability of two older names, *Hellenia* Retz. and *Tsiana* J.F.Gmel., both equally eligible for the clade of Asian Costaceae and proposed a new generic name, *Cheilocostus*. Govaerts (2013), while trying to fix the above mistake, unfortunately also overlooked the equally available *Tsiana* and proposed to use *Hellenia* Retz., without realising that it may very easily be confused with *Hellenia* Willd. which, although illegitimate, was used in the sister family Zingiberaceae for numerous species currently subsumed under *Alpinia* Roxb. Leong-Škorničková and Šída (2016), therefore, put forward a proposal to conserve *Cheilocostus*, which, since 2006, quickly gained very wide usage. In line with our proposal, we, therefore, continue to uphold the genus *Cheilocostus* until the decision by the Nomenclatural Committee of Vascular Plants is reached.

Only two Costaceae species are recorded to occur in Vietnam, the terminally flowering *Cheilocostusspeciosus* and the basally flowering *Costustonkinensis* (Phạm 2003). During our explorations of southern Vietnam, we have encountered an unusual radically flowering *Cheilocostus* with white flowers, which is described and illustrated below as *Cheilocostuscandidus*. We also designate the lectotype *Costustonkinensis* and effect its transfer into *Cheilocostus*. A key to the three species in Vietnam is provided.

**Figure 2.**
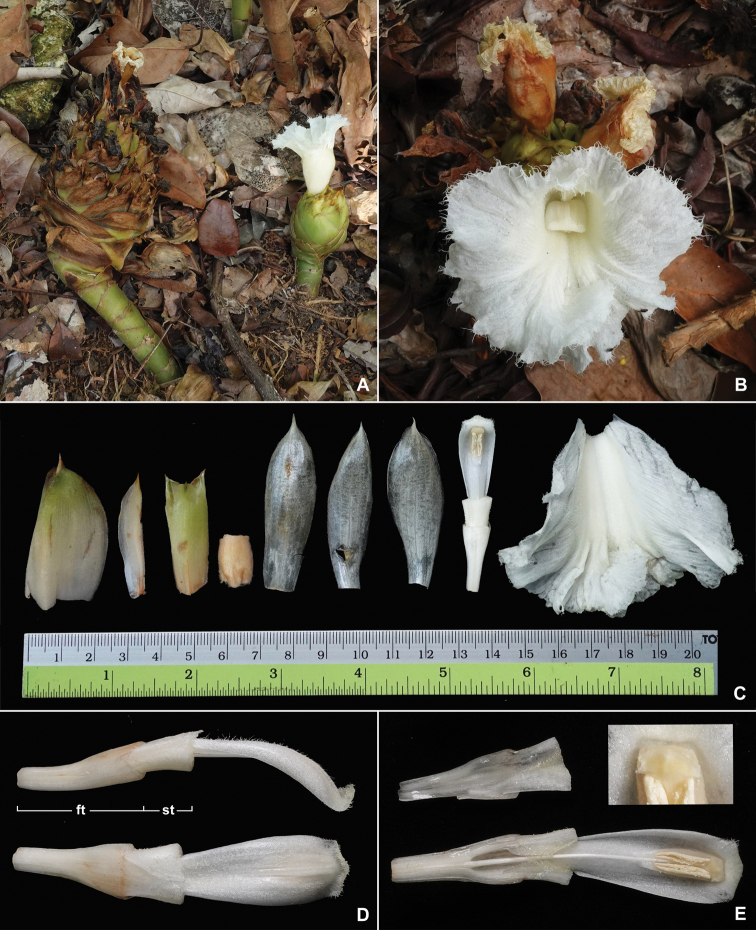
*Cheilocostuscandidus* Škorničk., Böhmová & H.Ð.Trần sp. nov. **A** old and young inflorescence **B** flower **C** flower dissection (from left): bract, bracteole, calyx, ovary, dorsal corolla lobe, two lateral corolla lobes, floral and staminal tube with stamen attached, labellum **D** detail of floral (ft) and staminal tube (st) with stamen attached from side and dorsal view **E** detail of floral and staminal tube dissected with stamen attached to dorsal half (inset: detail of stigma). Based on plant used to prepare the type specimen *Leong-Škorničková GRC-421*, photo by Jana Leong-Škorničková.

The description of the new species is based on measurements from living flowering material. The general terminology follows Beentje (2016). The style of the description largely follows Maas-van de Kamer et al. (2016) with the following amendments. We refrain from using the term corolla tube because this term is morphologically misleading. The tubular part of the flower is primarily formed by the fused bases of the members of androecium. In fact, only the most basal part of the tube, here referred to as the floral tube, is formed by fusion/adnation of the basal part of corolla lobes (external part of the tube) and staminal whorl (internal part of the tube). After the corolla lobes diverge, the tube further extends proximally and is formed solely by the staminal whorl (i.e. labellum and stamen) and is, therefore, termed here as the staminal tube (see Fig. 2D). This usage is in line with terminology used in the sister family Zingiberaceae, particularly (e.g. Leong-Škorničková 2007, Leong-Škorničková et al. 2016). In line with the explanation provided by Newman and Kirchoff (1992), we also use the term gynopleural nectaries instead of septal nectaries.

## ﻿Taxonomic treatment

### ﻿ Cheilocostus candidus

Taxon classificationPlantaeZingiberalesCostaceae

Škorničk., Böhmová & H.Ð.Trần sp.nov.

1848382C-0BE0-5AC8-B2DB-1663B0F70714

urn:lsid:ipni.org:names:77260720-1

Figures 2 , 3

#### Diagnosis.

Similar to *Ch.tonkinensis* by its inflorescence appearing at the base of the leafy shoots, but differs by its unbranched or very sparsely branched stems, densely puberulent leaf sheaths, densely puberulous lower side of lamina and cream-white flowers (compared to densely branched stems, glabrous leaf sheaths, glabrous lamina on both sides and yellow flowers with red markings on basal part of the labellum).

**Figure 3.**
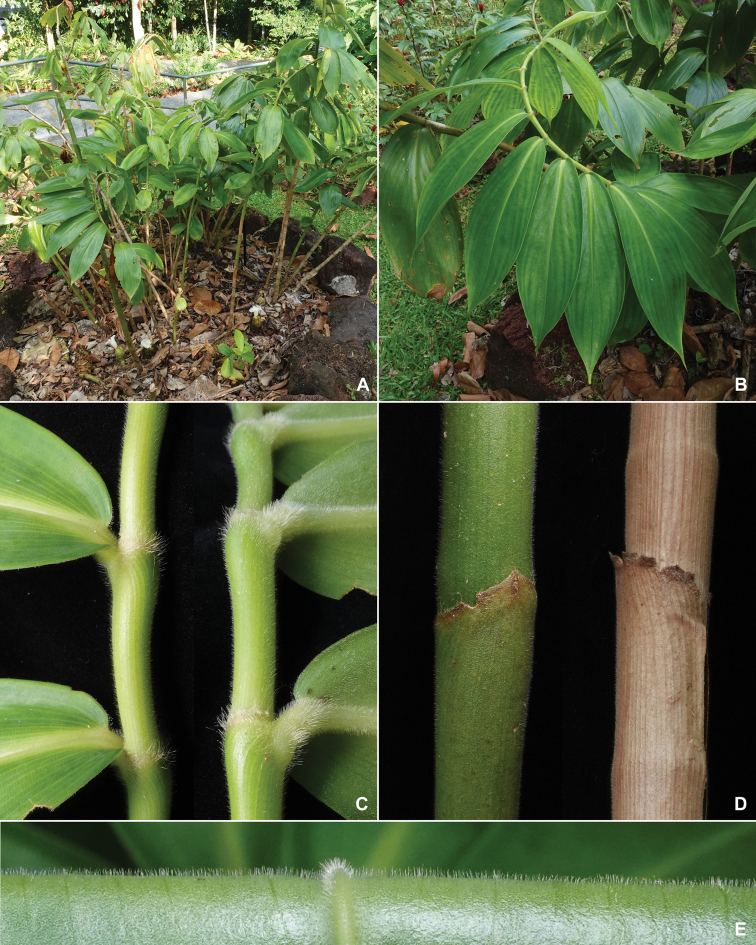
*Cheilocostuscandidus* Škorničk., Böhmová & H.Ð.Trần sp. nov. **A** habit **B** leaves **C** detail of young stem with pulvini and bases of leaf blades **D** detail of young and old stems covered with leaf sheaths **E** detail of lower surface of the leaf blade. Based on plant used to prepare the type specimen *Leong-Škorničková GRC-421*, photo by Jana Leong-Škorničková.

#### Type.

Collected from the material cultivated at the Singapore Botanic Gardens, 21 September 2021, *Leong-Škorničková GRC-421* (holotype SING (inclusive flowering material in spirit), isotypes E, P, SGN). Originally collected from Vietnam, Lâm Đồng Prov., 20 June 2008, *Trần et al. 54* (living material only).

#### Description.

Terrestrial, perennial herb in loose small clumps to ca. 2 m tall; **stems** up to 2.5 cm in diam. at base, unbranched or barely branched (in stems where the apical part was damaged) with 15–35 leaves, leafless in lower third; **sheaths** green, puberulent, becoming brown and papery with age; **ligule** 1–2 mm long, irregularly truncate, pale green, becoming pale brown with age, puberulous; **petiole** 2–5 mm long, pale green, bluntly canaliculate (kidney-shaped in cross-section), pubescent; **lamina** weakly obovate to elliptic with round to obtuse, slightly unequal base and acuminate apex, 12–28 × 4–11 cm, weakly plicate, somewhat thick, adaxially mid-green, glabrous, abaxially paler green, puberulous. **Inflorescence** radical, on a separate leafless shoot emerging directly from the rhizome; **peduncle** horizontal to ascending, 3–12 cm long, sheaths ± tubular, green, turning brown with age, densely pubescent, margin irregular; **spike** ovoid to narrowly ovoid, up to 20 cm long, 4.5–8 cm wide (narrower in very young, widening as the flowering progresses), composed of up to ca. 120 imbricate bracts, arranged in right-handed spiral, each supporting a single bracteole and single flower (lowermost 5–10 sterile), 1–2 flowers per inflorescence open at a time; **fertile bracts** ovate to broadly ovate, to 3.2–5.5 × 2.5–3.5 cm, cream at base, light green distally, softly pubescent externally, glabrous internally, apex ending in very sharp callus, callus 6–7 mm long, glabrous; **bracteole** 3–4 × 0.9–1.2 cm long, unequally folded with single sharp keel, white to cream at base, pale green distally, externally softly pubescent, internally glabrous, apex acuminate with very sharp callus, callus ca. 5–7 mm long. **Flower** 9–11 cm long, exserted 5.5–7 cm above the supporting bract; **calyx** 3.5–4 cm long, cream to pale beige at base, light green distally, densely pubescent, apex 3-lobed, lobes 5–10 mm long, ending with very sharply mucronate callus, each callus ca. 4–5 mm long, glabrous; **floral tube** (measured from the apex of ovary to the point of divergence of corolla lobes) 2–2.5 cm long, fused solid with style in basal ½, white to cream, externally and internally glabrous; **staminal tube** (measured from the point of divergence of corolla lobes to the point of divergence of stamen form the labellum) 8–11 mm long, white to cream, externally glabrous or with sparse hair, internally with long glandular hair; **corolla lobes** unequal, translucent cream-white, densely pubescent externally, glabrous internally, apex mucronate, mucro to 2–5 mm long, dorsal lobe obovate, symmetric, 5.1– 5.8 × 1.9–2.2 cm, lateral lobes unequal in width, asymmetrically obovate, 4.8–5.2 × 1.7–2 cm; **labellum** 5.5–6 cm long, ca. 7–8 cm broad (when flattened), broadly obovate with apex often bilobed (lobes overlapping), thickened in the centre, thin towards the margins, cream white to very pale yellow in the central part, adaxially with glandular hair (more dense in central part, nearly glabrous towards the margins), abaxially mostly glabrous with some glandular hairs, margin crisped, with glandular hair; **stamen** petaloid, slightly obovate, ca. 2.6 cm long (ca. 3 cm with crest flattened), ca. 6 mm wide at base, ca.1.3 cm wide at widest point, white to cream-white with pale yellow crest, adaxially glabrous, abaxially with sparse long glandular hair, crest 3–4 mm long, ca. 10 mm wide at base, rounded to obtuse, recurved, with glandular hair at margin; thecae 9–10 mm long, 3–4 mm across (both), dehiscing throughout their entire length. **Ovary** barrel-shaped, 13–16 × 10 mm, cream covered with soft dense beige hair, somewhat flattened and irregularly triangular in cross-section, trilocular with central placentation and apically embedded cream to beige coloured gynopleural nectaries, each locule with many ovules; **style** ca. 4 cm long (free part), white, glabrous; **stigma** semi-circular, 2 mm long, ca. 4 mm wide, dorso-ventrally flattened, 2-lamellate with dorsal 2-lobed appendage, cream white. **Fruits and seeds** not seen.

#### Etymology.

The specific epithet refers to the white colour of the flower.

#### Distribution.

Endemic to Vietnam.

#### Habitat and phenology.

This species occurs near rocky streams in lowland broadleaved evergreen forest, at elevations about 200–300 m. Flowering in the field was observed in June, in cultivation, it extends to October; fruiting has not been so far observed.

#### Provisional IUCN assessment.

The species is, so far, known only from the type locality, where only a few individuals were seen. A suitable habitat exists in the proximity of the type locality and it is, therefore, most likely that the species has a wider area of distribution as it is highly unusual for Costaceae species to be stenoendemic. Nevertheless, as there is no reliable information on the population sizes or distribution of this species, we propose to treat it currently as Data Deficient (IUCN 2019).

#### Notes.

*Cheilocostuscandidus* is similar to *Ch.tonkinensis* in producing the inflorescence radically. It is, however, fairly easy to recognise it in the field even in sterile conditions by its barely branched leafy shoots, which have densely puberulent leaf sheaths and densely puberulous lower surface of lamina, compared to multi-branched stems and always glabrous leaves of *Ch.tonkinensis*.

Axillary branching of the leafy stems has been mentioned as one of the generic descriptions of *Cheilocostus* by Specht and Stevenson (2006). Since then, *Ch.borneensis* A.D. Poulsen, a species that does not have axillary branching has been described (Poulsen and Specht 2010)). *Cheilocostuscandidus* also does not branch or branches very sparsely (usually only after extensive damage of the main shoot by herbivory or cutting), which is yet another character helpful in the field.

No other specimens of this species were found in E, HN, K, P, SING, and VNM.

### ﻿ Cheilocostus tonkinensis

Taxon classificationPlantaeZingiberalesCostaceae

(Gagnep.) Škorničk. comb. nov.

05D54718-30CD-5EAC-929E-5FFDE99ACC32

urn:lsid:ipni.org:names:77260721-1

Figure 1B

Costus tonkinensis Gagnep., Bull. Soc. Bot. France 49: 248. 1903 [1902 publ. 1903], Type: VIETNAM, Mount Ba Vì, *Balansa s.n.*, October 1887 (lectotype P! [P00686610], here designated).

#### Notes.

The type locality of *Cheilocostustonkinensis* is in northern Vietnam, Mount Ba Vì. In the protologue, Gagnepain (1902) refers to two collections made by Balansa from this locality. There are four sheets at P: a single sheet of *Balansa s.n.*, collected on 24 July 1886 [P00686610 – inflorescences & flower] and three sheets of *Balansa 4206* collected in October 1887 [P02198384 – leafy shoot & single fruit; P02198381-inflorescence only; P02198385-leafy shoot only]. Both of these collections predate the protologue and are annotated in Gagnepain’s hand as published in Bull. Bot Soc. France 49: 248. All four sheets have to be considered as syntypes and, therefore, the lectotypification is needed. Unfortunately, none of the specimens consists of both leafy shoot and an inflorescence. *Balansa s.n.* [P00686610] with inflorescence and a flower, accompanied by a pencil drawing, is selected here as the lectotype.

It is challenging to distinguish Asian radically flowering Costaceae in herbarium material as the flowers do not preserve well. Based on our observations of living flowering material and photographic records with location data, *Cheilocostustonkinensis*, so far, occurs in S. China, northern and central Vietnam and northern Laos, but may possibly extend to Thailand.

### ﻿Key to *Cheilocostus* in Vietnam

**Table:** 

1	Inflorescence arising on the top of the leafy shoot, labellum white	***Ch.speciosus***
–	Inflorescence arising from the base of the leafy shoot	**2**
2	Plants well-branched, sheaths, petioles, and both surfaces of laminae glabrous, labellum yellow, with red marking in the centre	***Ch.tonkinensis***
–	Plants sparsely or not branched, sheaths and abaxial surface of petioles and laminae puberulent to puberulous, labellum cream white throughout	***Ch.candidus***

## Supplementary Material

XML Treatment for Cheilocostus candidus

XML Treatment for Cheilocostus tonkinensis
